# Correlation properties and respiratory frequency of ECG‐derived heart rate variability during multiple race‐pace running intervals in female and male long‐distance runners

**DOI:** 10.14814/phy2.70177

**Published:** 2025-02-04

**Authors:** Thomas Gronwald, Marcelle Schaffarczyk, Dominik Fohrmann, Olaf Hoos, Karsten Hollander

**Affiliations:** ^1^ Institute of Interdisciplinary Exercise Science and Sports Medicine MSH Medical School Hamburg Hamburg Germany; ^2^ G‐Lab, Faculty of Applied Sport Sciences and Personality BSP Business and Law School Berlin Germany; ^3^ Center for Sports and Physical Education, Faculty of Human Sciences Julius‐Maximilians‐University Wuerzburg Wuerzburg Germany

**Keywords:** autonomic nervous system, DFAa1, exercise prescription, HRV, running economy

## Abstract

Aim was to evaluate alterations of the non‐linear short‐term scaling exponent alpha1 of detrended fluctuation analysis (DFAa1) of heart rate (HR) variability (HRV) as a sensitive marker for assessing global physiological demands during multiple running intervals. As a secondary analysis, agreement of ECG‐derived respiratory frequency (EDR) compared to respiratory frequency (RF) derived from the metabolic cart was evaluated with the same chest belt device. Fifteen trained female and male long‐distance runners completed four running bouts over 5 min on a treadmill at marathon pace. During the last 3 min of each bout gas exchange data and a single‐channel ECG for the determination of HR, DFAa1 of HRV, EDR and RF were analyzed. Additionally, blood lactate concentration (BLC) was determined and rating of perceived exertion (RPE) was requested. DFAa1, oxygen consumption, BLC, and RPE showed stable behaviors comparing the running intervals. Only HR (*p* < 0.001, *d* = 0.17) and RF (*p* = 0.012, *d* = 0.20) indicated slight increases with small effect sizes. In addition, results point towards inter‐individual differences in all internal load metrics. The comparison of EDR with RF during running revealed high correlations (*r* = 0.80, *p* < 0.001, ICC_3,1_ = 0.87) and low mean differences (1.8 ± 4.4 breaths/min), but rather large limits of agreement with 10.4 to −6.8 breaths/min. Results show the necessity of EDR methodology improvement before being used in a wide range of individuals and sports applications. Relationship of DFAa1 to other internal load metrics, including RF, in quasi‐steady‐state conditions bears the potential for further evaluation of exercise prescription and may enlighten decoupling mechanisms during prolonged exercise bouts.

## 1 INTRODUCTION

Analyses of the non‐linear characteristics of heart rate (HR) variability (HRV) indicate that the short‐term scaling exponent alpha1 of detrended fluctuation analysis (DFAa1) may be a sensitive marker for assessing global physiological demands during endurance exercise (Gronwald & Hoos, 2020; Gronwald, Rogers, & Hoos, 2020; Rogers & Gronwald, 2022). DFAa1 quantifies the fractal scale and correlation properties of HR time series in cardiac beat‐to‐beat intervals and represents a rather qualitative marker of autonomic nervous system regulation. Given these properties, and considering the corresponding signal‐theory background, this metric may be used as a biomarker for exercise intensity domain delineation (Gronwald, Rogers, & Hoos, 2020). For this purpose, it could be shown, that discrete numerical values of DFAa1 may demarcate the transition from moderate to heavy exercise intensity (DFAa1 of 0.75) and from heavy to severe exercise intensity (DFAa1 of 0.5) (3‐zone‐model), and may correspond to traditional threshold markers based on different physiological subsystem measures like blood lactate concentration (BLC) or gas exchange data (Mateo‐March et al., 2023; Rogers, Giles, Draper, Mourot, & Gronwald, 2021; Schaffarczyk et al., 2023; Sempere‐Ruiz et al., 2024; Van Hooren, Mennen, et al., 2023). Further, DFAa1 has been shown to be useful as an additional marker of acute fatigue in terms of systemic perturbation patterns in HR time series (Nuuttila et al., 2024; Rogers, Giles, Draper, Hoos, & Gronwald, 2021; Schaffarczyk et al., 2022; Van Hooren, Bongers, et al., 2023; Van Hooren, Mennen, et al., 2023) or as a measure of fatigue resistance in studies with prolonged exercise (Gronwald et al., 2018, 2019, 2024; Gronwald, Berk, et al., 2021; Nuuttila et al., 2024). Therefore, expanding these findings to future approaches of real‐time monitoring of steady‐state exercises typically used in training sessions seems to be promising, as the DFAa1 marker might bear the potential to mirror decoupling mechanisms as alterations of external‐to‐internal‐load relationships (Maunder et al., 2021; Smyth et al., 2022).

In this context, respiratory frequency (RF) was recently endorsed as a promising internal load marker for intensity monitoring and acute performance decrement during endurance exercise as well, with new possibilities for wearable analyses in research and practical settings (Nicolò et al., 2017, 2020; Nicolò & Sacchetti, 2023; Passfield et al., 2022; Tipton et al., 2017). Currently, there is a large interest in exercise science and sports practice to analyze RF via wearable technology and remote devices (Vitazkova et al., 2024) and especially to further evaluate typical endurance training sessions in combination with HR‐based measures (Seiler, 2024).

Data of DFAa1 and estimated RF derived from an electrocardiogram (ECG‐derived RF; EDR; Rogers, Schaffarczyk, & Gronwald, 2022) bear the potential of a more comprehensive internal load assessment during endurance exercise with real‐time applications recorded with a chest belt form factor complementary to established internal load indicators like HR and rating of perceived exertion (RPE). However, data on DFAa1 and estimated RF via EDR during steady‐state exercise bouts are scarce, and the true significance of exercise prescription remains to be elucidated. This applies especially to data during running exercises, given the high risk of movement artifacts and signal distortion in ECG‐waveform and HRV analysis (Aström et al., 2003). In addition, it has to be considered that the interaction between RF and autonomic nervous system regulation during exercise, mainly reflected in respiratory sinus arrhythmia, is a rather complex construct of an integrated interaction of a central‐medullary respiratory rhythm, a neural, extracardiac factor, and a non‐neural, intracardiac factor (cardiac mechano‐electrical feedback) (Kohl & Ravens, 2003). In this context, ‘respiratory gating’ of autonomic nervous system control (Eckberg, 2003), intrathoracic pressure changes (Cheyne et al., 2020), and direct cardiac mechano‐electrical feedback (Kohl & Ravens, 2003) interact in a complex, non‐linear way depending on exercise intensity and may therefore challenge the direct link between RF and different HRV derived metrics of autonomic nervous system function.

Therefore, the aim of the present report was to evaluate alterations of DFAa1 compared to further respiratory and metabolic measures, including EDR and actual measured RF via metabolic cart, during multiple intervals of running at marathon race pace in a group of trained female and male long‐distance runners. Our hypothesis was that DFAa1 and RF, as prescription parameters, do not differ significantly between running intervals and can potentially be used to provide feedback on the internal load situation during running with constant speed.

## 2 METHODS

### 2.1 Participants

Fifteen trained marathon (5 m, 3w) and half‐marathon (3 m, 4w) runners (age: 32.6 ± 5.4 years, body height: 174.6 ± 7.6 cm, body weight: 64.5 ± 7.8 kg) were recruited from the German athletics federation, Hamburg athletics federation and from local clubs through personal contacts during September and December 2023. Inclusion criteria were race performance in the marathon and half‐marathon corresponding to 400 points in the World Athletics “Scoring Table of Athletics” (Spiriev, 2022), age between 18 and 65 years, and absence of injuries >3 months before measurements. Ethical approval for the present study was given by the local ethics committee of the MSH Medical School Hamburg (reference no.: MSH‐2023/233). All participants gave written informed consent and all testing and measurements were conducted in accordance with the principles of the recent revision of the Declaration of Helsinki.

### 2.2 Study design

The cross‐sectional assessment was part of a larger study that aimed to investigate running economy and habituation with advanced footwear technology (Fohrmann et al., 2024; Schwalm et al., 2024). Based on the initial study setting, with a single laboratory session, participants completed four to six running bouts over 5 min at submaximal velocity based on individual running speed in marathon or half‐marathon. Running speed was defined as the pace of the marathon season's best or converting the season's best race performance in the half‐marathon into an estimated marathon time and corresponding race speed (multiplied by the factor of 2.11 from Steffny, 2016). The first four running bouts were used for the present study analysis.

### 2.3 Data recording

**FIGURE 1:**
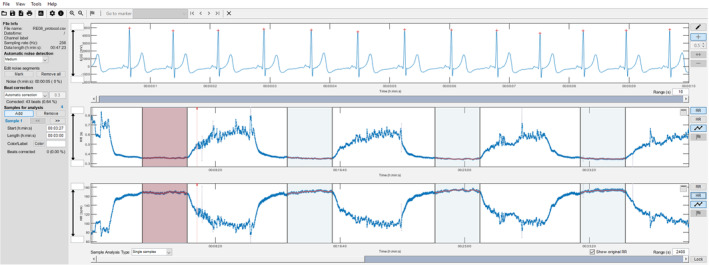
Course of an example of a single‐channel ECG tracing and corresponding RR‐intervals and HR of the running session with four running bouts over 5 min at submaximal velocity corresponding to individual running speed in marathon of one participant. The red shaded area indicates the analysis interval over 3 min of the first running bout designated as a specific warm‐up at race speed; the blue shaded areas indicate the analysis intervals of the second, third and fourth running bout. Screenshot modified from Kubios HRV Premium (version 3.5.0).

Body height and body weight of the participants were assessed using an analysis scale (655‐US, seca GmbH & Co. KG., Hamburg, Deutschland). In addition, participants were asked for their maximum HR (HR_MAX_) from a recent treadmill performance test or competition. In case of unknown maximum HR calculation according to Tanaka's formula was applied: 208–0.7 x age (Tanaka et al., 2001). Afterwards, a general warm‐up over 10 min was conducted at preferred running speed prior to the running bouts at submaximal velocity on a motorized treadmill over 5 min (FDM‐T, h/p/cosmos, Nussdorf‐Traunstein, Germany); the first bout of the intervention trials was designated as a specific warm‐up at race speed (see Figure 1). Immediately after the running bouts BLC (in mmol/L) from the capillary blood of the earlobe (20 μL) with the Biosen C‐Line Clinic analyzer (EKF‐diagnostic GmbH, Barleben, Germany) was determined and RPE was requested using the Borg scale (6–20; Borg, 1982). A passive break of 5 min was introduced in between the running bouts. Recordings of a single‐channel ECG for the determination of HR (in beats per minute, bpm), RR‐intervals (in ms) and EDR (in breaths/min) were taken continuously with the Movesense Medical sensor (firmware version 2.1.2) implemented in a chest belt (Movesense, Vantaa, Finland) and the Movesense Showcase app via smartphone (sampling rate: 256 Hz; iOS: version 1.1.0; Rogers, Schaffarczyk, Clauß, et al., 2022, see Figure 1). Breath‐by‐breath pulmonary gas exchange data were recorded using a metabolic cart (Quark CPET, module A‐670‐100‐005, COSMED Deutschland GmbH, Fridolfing, Germany; Omnia version 2.2). Expired gas fractions were continuously measured to determine oxygen consumption (VO_2_, in mL/min/kg), RF (in breaths/min), and minute ventilation (VE, in L/min). All physiological measures were determined during the last 3 min of each running bout. Resting values were taken prior to the general warm‐up period over 2 min.

### 2.4 HRV and EDR analysis

To analyze HR, RR‐intervals and EDR data were exported from the Movesense Showcase app via .csv file and processed in Kubios HRV Premium (version 3.5.0, Biosignal Analysis and Medical Imaging Group, Department of Physics, University of Kuopio, Kuopio, Finland). Preprocessing settings were set to the default values, including the RR detrending method, which was kept at “smoothness priors” (Lambda = 500). The RR‐interval series were then corrected using the Kubios HRV “automatic correction” method (Lipponen & Tarvainen, 2019). HR and DFAa1 were determined during the last 3 min of each running bout. To calculate DFAa1, the root mean square fluctuations of the integrated and detrended RR‐intervals were analyzed in observation windows of different sizes and then further processed as the slope between the root mean square fluctuation data in relation to the different window sizes on a log–log scale (Peng et al., 1995). Window size was set to 4 ≤ *n* ≤ 16 beats in the software preferences. For EDR assessment Kubios HRV software RF estimation algorithm was used (Lipponen & Tarvainen, 2021). The algorithm combines the cyclic cardiac beat‐to‐beat time domain changes in RR intervals associated with respiratory sinus arrhythmia and the single‐channel ECG‐associated R wave amplitude changes seen during the respiratory cycle. For EDR calculation, the window width was set to 30 s with a recalculation grid interval of 1 s based on recommendations from Lipponen and Tarvainen (2021). During the last 3 min of each running bout, maximum EDR was noted. Data sets with >5% artifacts were excluded from RR‐interval and EDR analysis. Data were also scanned visually for artifacts and ECG tracing quality by an expert with experience in HRV‐data analysis and removed manually if necessary.

### 2.5 Efficiency factor

For the analysis of internal‐to‐external‐load relationship and a possible decoupling mechanism in comparison of the running bouts an efficiency factor (EF) was defined. This internal‐to‐external workload ratio was calculated using the ratio of the internal load indicators (VO_2_, RF, VE, BLC, RPE, HR, %HR_MAX_, DFAa1, and EDR) and running pace (in km/h). The difference of the EF between the second and the fourth running bout was calculated and divided by the EF from the second running bout multiplied by 100 to get a percentage of alteration (%). Thus, for example, a value of 10% indicates that internal‐to‐external ratio was 10% greater during the fourth running bout compared to that observed in the second running bout (Maunder et al., 2021; Smyth et al., 2022).

### 2.6 Statistical methods

The statistical analysis was performed using SPSS (version 27.0, IBM Statistics, USA) for Windows (Microsoft, USA), RStudio (version 2023.12.1, RStudio Team, Boston, MA, USA), and Microsoft Excel (Microsoft Corp, Redmond, USA). The Shapiro–Wilk test was applied to verify the Gaussian distribution of the data. The degree of variance homogeneity was verified by Levene test. Subsequently, linear mixed modeling incorporating within‐person factors was used to evaluate physiological changes over time (for data of second, third, and fourth running intervals). Paired t‐tests were applied to analyze differences between the second and the fourth running bout. In addition, Cohen's *d* was calculated for effect size estimation (difference between mean values divided by the pooled standard deviation) with no effect (*d* < 0.2), small effect size (0.2 ≤ *d* < 0.5), moderate effect size (0.5 ≤ *d* < 0.8) and large effect size (*d* ≥ 0.8) (Cohen, 1988). Further, interrelations and agreement between RF and EDR of all available data pairs of the rest condition, and all running bouts were evaluated using either linear regression with Pearson's correlation coefficient (*r*) and coefficient of determination (*R* ^2^), or repeated measures correlation (r_rm_, Bakdash & Marusich, 2017), Intraclass Correlation Coefficient (ICC_3,1_), and Bland–Altman plot with limits of agreement (LoA) (Bland & Altman, 1999), adjusted for repeated measures (Parker et al., 2016). In addition, the mean absolute error (MAE) was calculated as the sum of absolute errors divided by the number of available data pairs of the rest condition, and all running bouts to add a quantification of the mean random scattering around the systematic bias (mean difference) and to account for different directions of this difference. If proportional bias was detected (change in the bias over the RF range), a regression‐based calculation of mean differences was performed (Ludbrook, 2010). The size of Pearson's *r* correlation coefficient was evaluated as follows: low: 0.3 ≤ *r* < 0.5; moderate: 0.5 ≤ *r* < 0.8, high: *r* ≥ 0.8 (Chan, 2003). Bland–Altman mean differences for data comparisons were expressed as absolute bias. The paired t‐test was used for comparison of RF vs. EDR. Statistical tests were deemed to be significant at *p* ≤ 0.05. All results are reported as means ± standard deviation (SD).

## 3 RESULTS

The season's best times corresponded to 795.7 ± 246.0 points of the World Athletics “Scoring Table of Athletics” (Spiriev, 2022), related to mean marathon times of 2:41:20 h:min:s and mean half‐marathon times of 1:26:40 h:min:s. Consequently, mean running speed for the four submaximal running bouts was 15.3 ± 2.4 km/h (MIN: 11.7 km/h, MAX: 19.5 km/h). Linear mixed modeling revealed significant main effects of time for RF, HR, and %HR_MAX_ (VO_2_: *F* = 0.224, *p* = 0.801; RF: *F* = 6.818, *p* = 0.004; VE: *F* = 0.509, *p* = 0.606; BLC: *F* = 0.279, *p* = 0.759; RPE: *F* = 0.596, *p* = 0.558; HR: *F* = 12.522, *p* < 0.001; %HR_MAX_: *F* = 12.757, p < 0.001; DFAa1: *F* = 0.267, *p* = 0.768; EDR: *F* = 0.374, *p* = 0.693). In comparison of the second and fourth running bout both RF and HR showed statistically significant increases with small effect sizes. Furthermore, EF revealed values <5% for all internal load indicators (see Table 1).

**TABLE 1:** Physiological measures during resting state before and during the four running bouts: Mean ± SD (Range: min–max).

Measure	Rest	First bout (specific) warm‐up	Second bout	Third bout	Fourth bout	Statistics^a^
VO_2_ [mL/min/kg], *n* = 15	5.38 ± 0.92 (3.16–6.70)	49.34 ± 6.30 (39.30–61.87)	50.19 ± 6.53 (39.20–63.47)	50.04 ± 6.16 (40.10–64.51)	50.21 ± 6.59 (39.20–65.85)	*p* = 0.939, *d* = 0.00, EF = 0.1%
RF [breaths/min], *n* = 15	14.1 ± 3.7 (9.4–19.6)	41.5 ± 6.3 (33.3–56.1)	44.2 ± 6.5 (35.9–61.5)	44.5 ± 7.4 (34.2–63.0)	45.6 ± 7.4 (36.6–63.2)	*p* **= 0.012**, *d* = 0.20, EF = 3.1%
VE [L/min], *n* = 15	12.1 ± 4.4 (6.3–22.2)	86.6 ± 17.2 (61.6–109.0)	87.4 ± 16.1 (66.5–108.8)	88.2 ± 17.2 (63.8–111.9)	88.2 ± 16.4 (64.9–114.7)	*p* = 0.461, *d* = 0.05, EF = 1.0%
BLC [mmol/L], *n* = 15	1.27 ± 0.21 (1.02–1.76)	2.12 ± 0.78 (1.11–3.69)	2.09 ± 0.72 (1.29–3.82)	2.05 ± 0.85 (1.16–4.00)	2.11 ± 0.90 (1.26–4.07)	*p* = 0.862, *d* = 0.02, EF = −0.1%
RPE [6–20], *n* = 15	‐	12.8 ± 0.8 (11.0–14.0)	13.1 ± 0.9 (12.0–15.0)	12.8 ± 1.0 (11.0–14.0)	13.0 ± 1.3 (11.0–15.0)	*p* = 0.719, *d* = −0.15, EF = ‐0.7%
HR [bpm], *n* = 15	64.5 ± 9.7 (49.7–85.5)	163.0 ± 12.7 (145.5–190.5)	167.2 ± 12.5 (149.2–193.0)	168.4 ± 12.7 (151.0–195.5)	169.4 ± 13.0 (151.4–198.5)	*p* **< 0.001**, *d* = 0.17, EF = 1.3%
%HR_MAX_, *n* = 15	34.2 ± 5.1 (25.8–43.2)	86.5 ± 6.8 (77.4–97.0)	88.8 ± 6.5 (79.9–98.9)	89.4 ± 6.8 (79.0–99.5)	89.9 ± 6.7 (79.3–99.9)	*p* **< 0.001**, *d* = 0.18, EF = 1.3%
DFAa1, *n* = 11–14	1.03 ± 0.16 (0.70–1.21)	0.54 ± 0.26 (0.25–0.99)	0.54 ± 0.27 (0.26–0.93)	0.53 ± 0.25 (0.25–0.90)	0.51 ± 0.23 (0.18–0.87)	*p* = 0.585, *d* = −0.14, EF = 2.1%
EDR [breaths/min], *n* = 10–12	15.0 ± 4.1 (9.8–19.7)	39.0 ± 7.6 (23.6–55.8)	42.3 ± 7.0 (32.0–55.7)	42.6 ± 5.6 (33.6–56.3)	42.6 ± 4.0 (39.1–52.5)	*p* = 0.443, *d* = 0.04, EF = 1.7%

Abbreviations: %HR_MAX_, Percentage of maximum heart rate; BLC, Blood lactate concentration; DFAa1, Short‐term scaling exponent alpha1 of detrended fluctuation analysis; EDR, ECG‐derived estimated respiratory frequency; EF, Efficiency factor; HR, Heart rate; RF, Respiratory frequency; RPE, Rating of perceived exertion; VE, Minute ventilation; VO_2_, Oxygen consumption.

^a^ Comparison of the second and the fourth running bout.

Bold indicates highlighting significant changes.

**FIGURE 2:**
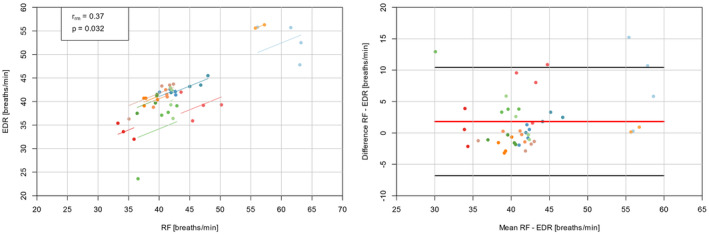
Repeated measures correlation (r_rm_) plot for all data pairs of the exercise bouts of RF vs. EDR values (left). Bland–Altman plot adjusted for repeated measures for all data pairs of the exercise bouts of RF vs. EDR values (right); center line in red represents the mean difference between each paired value, the top and bottom lines in black display ±1.96 standard deviations from the mean difference. Each color represents one participant.

Regarding the comparison of RF vs. EDR, 59 of 75 (79%) of all data pairs (resting condition, all four running exercise bouts) could be used. A strong linear relationship could be seen between the two measurement principles, with a high Pearson's *r* coefficient for the resting condition (*r* = 0.81, *p* < 0.001) and exercise bouts (*r*_rm_ = 0.37, *p* = 0.032), and an intraclass correlation coefficient ICC_3,1_ of 0.90 for the resting condition and 0.87 for the exercise bouts. The comparison of RF vs. EDR revealed no significant difference for resting data (*p* = 0.435, *d* = −0.13) but a significant difference for the exercise data (*p* = 0.008, *d* = 0.27). Bland–Altman analysis showed a mean difference of 1.3 ± 4.1 breaths/min (resting values: −0.5 ± 2.4; exercise values: 1.8 ± 4.4) with limits of agreement of 9.3 to −6.8 breaths/min (resting values: 4.2 to −5.2 breaths/min; exercise values: 10.4 to −6.9 breaths/min), respectively (see Figure 2). The MAE indicated a value of 2.7 ± 3.3 breaths/min (resting values: 1.6 ± 1.8 breaths/min; exercise values: 3.1 ± 3.6 breaths/min).

## 4 DISCUSSION

The aim of the present report was to evaluate alterations of DFAa1 in addition to respiratory and metabolic measures during multiple running intervals at marathon pace in a group of trained female and male long‐distance runners. Furthermore, agreement of EDR compared to RF derived from the metabolic cart was evaluated during resting conditions and the running bouts.

Results show that DFAa1 values decreased from ~1.0 at rest to ~0.5 at marathon running pace with no alterations when comparing the exercise bouts, which indicates a loss of fractal dynamics and a change towards uncorrelated and random behavior (Hautala et al., 2003; Peng et al., 1995). This corresponds to data from a study with recreational runners performing a self‐paced marathon road race on an almost flat profile (Gronwald, Rogers, et al., 2021). DFAa1 as a dimensionless index of correlation properties of HR time series and complex regulation has shown the ability to reflect physiological demands compared to other internal load measures. It is assumed that the kinetics of DFAa1 during exercise is based on changes in autonomic modulation due to parasympathetic withdrawal, sympathetic activation, altered non‐neural factors, and the potential loss of interaction between the two branches of the autonomic nervous system with increased organismic demands (Persson, 1996; White & Raven, 2014). Interestingly, and similar to the study of Gronwald, Rogers, et al. (2021) DFAa1 displayed a rather large inter‐individual dynamic range during the evaluated running bouts, denoting possible fluctuations in internal load situation at race pace. In addition, intermittent positional bounce such as seen with running could have affected ECG‐waveform morphology due to shifts in the cardiac axis (Aström et al., 2003). External load prescription assumes that physiological responses are rather static (Jamnick et al., 2020; Maunder et al., 2021) and neglect the influence of internal and external factors leading to heterogeneity in exercise tolerance and physiological responses over time (e.g., personal or environmental factors, Gronwald, Törpel, et al., 2020; Meyler et al., 2023).

Percentage of HR_MAX_ during the exercise bouts reached values corresponding to the transition of heavy to severe exercise intensity domain in a 3‐zone‐model of intensity distribution for moderate, heavy, and severe exercise domain (“moderate” to “high” domain within a recent proposal and consensus statement in exercise intensity terminology from Bishop et al., 2024) with significant increase but very small effect. The same applies to RF with small effect size and no change in VO_2_. These changes are in line with our hypotheses and with the expectable difference in HR and VO_2_ kinetics during constant load exercise (e.g., Zuccarelli et al., 2018) leading to the assumption of a quasi‐steady‐state condition at marathon running pace. However, small differences in the slope of decoupling mechanisms for e.g. HR related and RF related measures can be expected as they are regulated by different aspects of increasing central demand induced by peripheral fatigue processes (Seiler, 2024). RPE showed mean values of ~13 with a range of 11–15 and no significant differences in comparison of the running bouts, showing inter‐individual variation across participants. Blood lactate concentration revealed values around 2 mmol/L with no alterations over time but also considerable inter‐individual differences below the point of what may be considered a maximal lactate steady‐state (range: 1.02 to 4.07 mmol/L; Perrey et al., 2003). A study by Santos et al. (2006) took blood lactate samples every 6 km in elite marathon runners during a 30 km race and showed values from 2.4 mmol/L at 6 km to 3.2 mmol/L at 30 km. In a marathon field study, blood lactate values of 4.0 mmol/L could be observed immediately after the race (Gronwald, Rogers, et al., 2021). Overall, the calculated EF assessing potential decoupling mechanisms of internal‐to‐external load relationship revealed values under 5% showing almost no alteration in all internal load metrics in comparison of the running intervals. Therefore, this ratio may bear great potential for assessing possible decoupling mechanisms during prolonged running exercise bouts in comparison to different exercise intensity domains (Gronwald et al., 2024).

The comparison of EDR with RF derived from the metabolic cart revealed high correlation coefficients with a low mean difference across all paired values including the resting condition and all running bouts; with higher values for MAE analysis. However, limits of agreement were relatively wide and the absolute divergences in breaths/min could be still clinically or practically relevant on an individual level depending on the field of application. These results were also confirmed in an analysis across the entire intensity spectrum using the same sensor technology (Rogers, Mourot, Doucende, & Gronwald, 2021). A possible confounding factor for EDR measurement during running could be that HRV is influenced by step frequency. This can lead to overlaps and signal distortions caused by cardio‐locomotor‐respiratory coupling (Bailón et al., 2013; Niizeki et al., 1993). Since the intensity of this coupling can vary individually (Hottenrott et al., 2020), it may explain the observed individual discrepancies in RF estimation based on ECG and/or RR‐interval data. Further questions need to be clarified about suitability of different subsystem parameters of internal load and an “optimal” and feasible real‐time monitoring approach for the control of exercise intensity (e.g., HR drift and the potential underestimation of RPE; Cartón‐Llorente et al., 2022). Here, a dimensionless, global, and systemic internal load indicator like DFAa1, in addition to RPE and RF as indicators of acute performance decrement (Passfield et al., 2022), might be used to detect ongoing compensatory mechanisms and “homeodynamic” regulation pattern and could provide the potential for exercise prescription and further investigation in prolonged running exercise of different intensities (Gronwald et al., 2024; Nuuttila et al., 2024; Rogers & Gronwald, 2022).

Interpreting the results of our study, a few limitations should be taken into account. Our study included a small sample size of 15 participants. The number of running intervals was limited and therefore transfer of the applied exercise prescription for typical durations of running training (e.g., >30 min) may not be appropriate, as these longer durations may show further decoupling in internal‐to‐external load relationships (Seiler, 2024). In addition, the included recovery bouts in the present study could further influence decoupling processes due to on‐ and off‐kinetics. The data of the present report show that DFAa1 is related to other internal load indicators at a fixed marathon running pace and stabilizes under quasi‐steady‐state conditions. In conjunction with commonly used objective and subjective measures such as HR, BLC and RPE, DFAa1 can be applied for exercise prescription and real‐time internal load feedback.

For EDR assessment Kubios HRV Premium software estimation algorithm was used based on the combination of the cyclic cardiac beat‐to‐beat time domain changes in RR‐intervals associated with respiratory sinus arrhythmia and the single‐channel ECG‐associated R wave amplitude changes seen during the respiratory cycle (Lipponen & Tarvainen, 2021). However, as already mentioned, the complex nature of respiratory sinus arrhythmia during running exercise that integrates mechanisms of ‘respiratory gating’ (Eckberg, 2003), intrathoracic pressure changes (Cheyne et al., 2020), and direct cardiac mechano‐electrical feedback (Kohl & Ravens, 2003) exhibits influence on HR and HRV and this may show substantial between‐participants variability that challenge EDR estimations. Another aspect that definitely affects the results of this estimation algorithm is the design to accommodate a wide range of applications, from short resting measurements to long‐term recordings and sports applications (e.g., not specifically to signal quality aspects of running exercise). Therefore, adapation of this algorithm specialized to endurance sports applications with different types of exercise would potentially enhance validity within the estimation of RF via EDR and narrow the range of upper and lower limits of agreement. In addition, approximately 25% (see Table 1) of data had to be excluded for non‐linear HRV and EDR analysis due to data quality and artifact rate, which can still affect the use in sport‐specific field conditions. As mentioned before, the cardio‐locomotor‐respiratory‐coupling in running and corresponding aliasing and signal distortion effects might be specific challenges that need to be refined in future advances in sensor technology and HRV signal analysis to further improve signal integrity and reliability. In that regard, EDR analysis, together with the assessment of DFAa1 bears the potential of a more comprehensive internal load assessment in post‐exercise and real‐time analysis based on simple, low‐cost chest belt recordings (Gronwald et al., 2024; Gronwald, Berk, et al., 2021).

## 5 CONCLUSION

DFAa1 is defined as an indicator of relative internal load and proxy of physiological demands. It showed no substantial alterations in comparison to multiple running intervals at marathon race pace in female and male long‐distance runners. The comparison of EDR with RF derived from the metabolic cart during running revealed high correlations and low mean differences, but rather large limits of agreement. This shows the necessity of further improvement of the methodology before being used in a wide range of individuals and different sports applications. In addition, further research and development of sensor technologies and analysis algorithms are needed to realize the benefits of the chest strap form factor in sports practice. The present report showed that a fixed external load based on marathon running pace implies considerable inter‐individual differences in all internal load metrics. In this context, the relationship of DFAa1 to other traditionally used internal load metrics in quasi‐steady‐state conditions bears the potential for further evaluation of exercise prescription in general and the enlightenment of decoupling mechanisms during prolonged exercise bouts.

## AUTHOR CONTRIBUTIONS

K.H., D.F., M.S., and T.G. conceived the study. T.G., O.H. and K.H. designed the research question. M.S. and D.F. conducted the experiments and data processing. T.G., M.S. and D.F. conducted data analysis and interpretation. T.G. drafted the manuscript. All authors provided critical comments on the manuscript, read, and approved the final version of the manuscript.

## FUNDING INFORMATION

This project was funded by the German Federal Institute for Sports Science (Bundesinstitut für Sportwissenschaft, BISp, grant number: ZMI4‐072001/23).

## CONFLICT OF INTEREST STATEMENT

The authors declare no conflicts of interest.

## ETHICS STATEMENT

Ethical approval for the present study was given by the local ethics committee of the MSH Medical School Hamburg (reference no.: MSH‐2023/233). All participants gave written informed consent and all testing and measurements were conducted in accordance with the principles of the recent revision of the Declaration of Helsinki.

## Data Availability

Data are available from the corresponding author on reasonable request.
